# Pyopericardium with cardiac tamponade caused by pyogenic liver abscess: a case report

**DOI:** 10.1186/s13256-024-05014-z

**Published:** 2025-01-08

**Authors:** Carolin Steffen, Josef Sägmüller, Dominique Schöneburg, Eva Göncz, Martin Möckel, Sascha Ott, Alexander Lavinius Ungur, Benjamin O ’Brien

**Affiliations:** 1 Charité – Universitätsmedizin Berlin, Department of Anesthesiology and Operative Intensive Care Medicine, Freie Universität Berlin and Humboldt-Universität zu Berlin, Berlin, Germany; 2 Department of Cardiac Anesthesiology and Intensive Care Medicine, Deutsches Herzzentrum der Charité – Medical Heart Center of Charité and German Heart Institute Berlin, Augustenburger Platz 1, 13353 Berlin, Germany; 3 Labor Berlin – Charité Vivantes GmbH, Subsidiary company of Charité – Universitätsmedizin Berlin, Sylter Str. 2, 13353 Berlin, Germany; 4 DZHK (German Centre for Cardiovascular Research), Partner Site Berlin, Department of Perioperative Medicine, St Bartholomew’s Hospital and Barts Heart Centre, West Smithfield, London, EC1A 7BE UK

**Keywords:** Pyopericardium, Pyogenic liver abscess, Cardiac tamponade, Case report

## Abstract

**Introduction:**

Purulent bacterial pericarditis is a potentially fatal disease with mortality rates reaching 100% if left untreated.

**Case presentation:**

We present the case of a 33-year-old Caucasian male patient who developed cardiac tamponade, most likely caused by a pyogenic liver abscess communicating with the pericardium. Treatment with antibiotics, extended sepsis therapy, and drainage of the abscess led to a full recovery.

**Conclusion:**

This report describes a rare but potentially fatal differential diagnosis of aortic dissection and serves as a reminder that lives abscesses can manifest unexpectedly. Clinical signs and symptoms of tamponade can be mistaken as sepsis. In this particular case, the combination of a septic abscess and tamponade caused by pyopericardium posed a diagnostic challenge.

## Introduction

Pyopericardium is a rare condition most often diagnosed in neonates and children but rarely described in adults. While the most frequently reported pathogen is Staphylococcus aureus, Gram-negative bacteria and fungi (Candida and Aspergillus species) are increasingly being reported in the recent literature [1].

Typically, a pyopericardium can occur by hematogenous route, direct inoculation, or extension from adjacent structures, for example, pyogenic liver abscess (PLA). The following invasive treatments for PLA-associated pericarditis have been reported: pericardiocentesis, surgical pericardial drainage or pericardiectomy combined with abscess drainage, and surgical abscess resection [2].

## Case presentation

A 33-year-old Caucasian male patient was brought to our emergency department after experiencing two syncopal episodes on the morning of admission.

The patient, employed as a mechanic, had a history of chronic back pain that had worsened in the last 5 months prior to admission. Consequently, a magnetic resonance imaging (MRI) scan was conducted 2 months earlier, revealing signs of colitis but no spine-associated pathology.

Then 6 weeks before admission the patient began experiencing chest discomfort, loss of appetite, and diarrhea, leading to a substantial weight loss of 16 kg. Upon admission, he had a BMI of 40 kg/m^2^. The patient, who had never been hospitalized before, was solely treated for hypertension with valsartan. He presented with tachycardia and a slight fever (37.8 °C and 38.0 °C on two measurements). Oxygen saturation was recorded at 94% on 4 L of oxygen, accompanied by an elevated respiratory rate. Cardiovascular examination revealed sinus tachykardia (160 beats per minute), hypotension (90/60 mmHg), and muffled heart sounds. A transthoracic echocardiography (TTE) revealed a significant pericardial effusion with a swinging motion of the heart within effusion and compression of the right atrium as a sign of pericardial tamponade.

Laboratory test results revealed elevated systemic inflammatory markers [leukocytes 39.6 nL, procalcitonin (pct) 6.24 µg/L, C-reactive protein (CRP) 186.8 mg/L].

For additional differential diagnostics, a computed tomography (CT) scan was conducted to eliminate the possibility of an aortic dissection. While the echocardiographically identified pericardial effusion was confirmed, further findings revealed a subphrenic and retrosplenic abscess with infiltration to the left liver lobe.

**Fig. 1:**
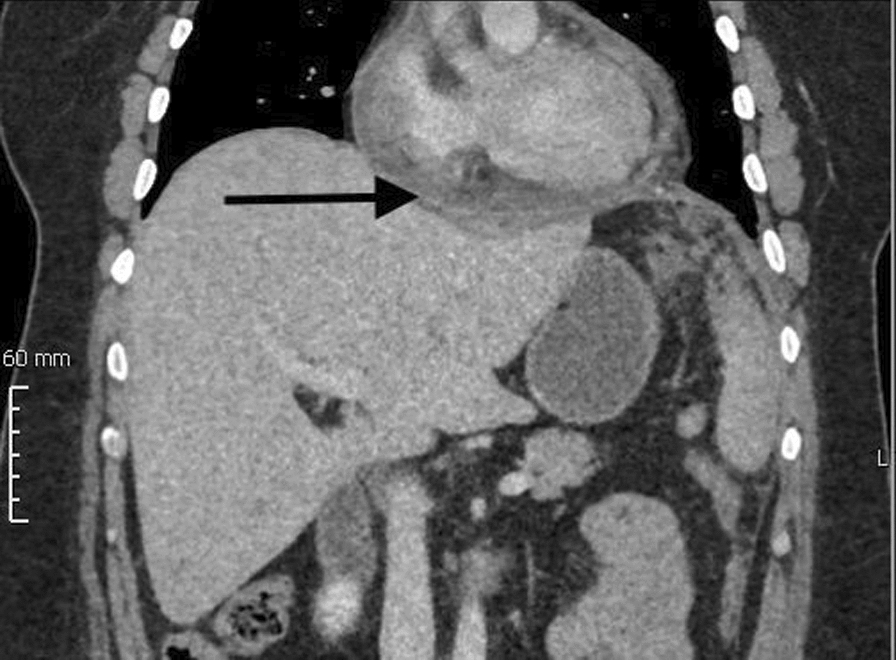
Computed tomography coronal plane images, black arrow points to transdiaphragmatic rupture of a large hepatic abscess into the pericardial space

The abscess extended transdiaphragmatically, connecting the pericardial effusion (Fig. 1). Subsequently, the patient was admitted to the intensive care unit.

Shortly after admission to the intensive care unit, the patient experienced rapid deterioration, manifesting as a combined septic and obstructive shock (lactate 9.32 mmol/l) leading to consecutive acute kidney injury (kreatinine 318.24 µmol/l). According to current sepsis guidelines, we promptly initiated blood sampling, broad antibiotic therapy, administered intravenous fluids, and conducted a CT-guided drainage of the abdominal and pericardial abscess cavities. Approximately 600 ml of purulent fluid was successfully drained. Within 48 hours, we observed hemodynamic stabilization, reduction in inflammation parameters, and complete resolution of the acute kidney injury. Coagulase-negative staphylococci (CoNS) were detected in only one sample of blood cultures, which was considered contaminated. Analysis of both pericardial and subphrenic drainage fluids revealed high concentrations of *Bacteroides fragilis*, *Hungatella hathewayi*, and *Fusobacterium nucleatum* (see Table 1 for antibiotic susceptibility). Due to previously mentioned signs of colitis in MRI, a feces analysis was performed and revealed no evidence of infectious diarrhea, including amebiasis.

**Table 1:** Minimum inhibitory concentration findings in mg/L

	Penicillin	Amoxicillin/clavulanic acid	Piperacillin/tazobactam	Imipenem	Clindamycin	Metronidazole
*Bacteroides fragilis*	0.19	0.064	0.125	0.032	0.125	0.25
*Hungatella hathewayi*	0.25	0.094	0.75	0.75	≤ 0.016	0.016
*Fusobacterium nucleatum subsp. vincentii*	≤ 0.016	≤ 0.016	≤ 0.016	0.002	≤ 0.016	≤ 0.016

As tuberculosis should be considered as an important differential diagnosis of liver abscess, a polymerase chain reaction (PCR) for Mycobacterium tuberculosis was conducted on abdominal drain fluid, confirming no evidence of tuberculosis.

As virulence factors help bacteria to invade the host, cause disease, and evade host defenses, a whole genome shotgun sequencing was performed, revealing no virulence factors when compared with an open-source database [3]. To eliminate the possibility of any other primary focus or predisposing enteric fistulae, we conducted a comprehensive examination, including gastroscopy, colonoscopy, transesophageal echocardiography, and an MRI of the spine and bile ducts.

Gastroscopy revealed mild gastritis without any indication of a fistula connecting the upper gastrointestinal tract to the pericardial area. Additionally, magnetic resonance cholangiopancreatography (MRCP) highlighted cholecystolithiasis but no sign of cholangitis, effectively ruling out further inflammation of the liver or the bile ducts.

**Fig. 2:**
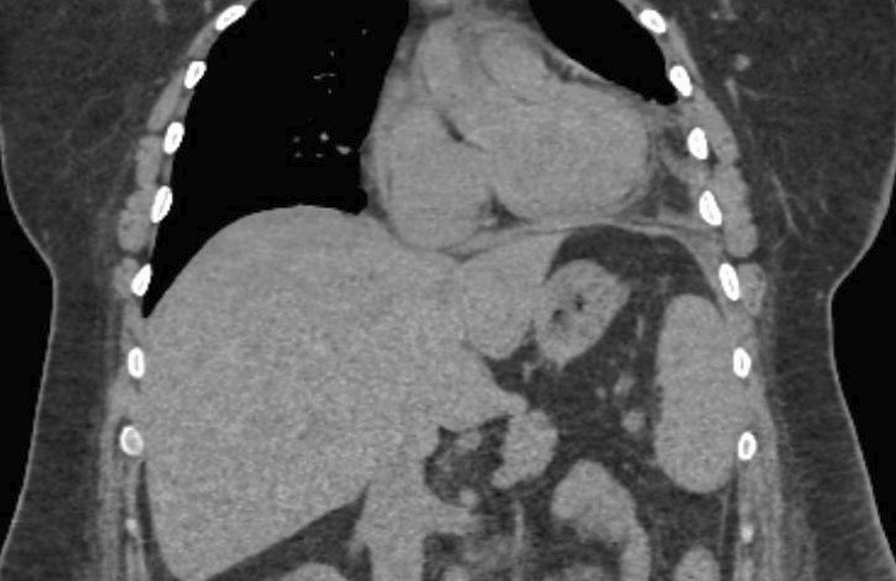
Computed tomography coronal plane images showing a regression of the pericardial effusion and decreased abscess formation

To ensure there was no ongoing communication between the abscess and the pericardial cavity, a CT scan was conducted 5 days after admission. Before the scan, a contrast agent was injected into the abdominal drain, which was still in place. The subsequent scan indicated no ongoing communication between the liver abscess cavity and the pericardium. Moreover, following CT scans showed decreasing pericardial effusion (Fig. 2.)

The patient fully recovered, was discharged from the intensive care unit (ICU) on day 14, and returned home on day 19 after admission. Late-onset complications, such as constrictive pericarditis, aortic mycotic aneurysm, or left ventricular pseudoaneurysm, were monitored through regular follow-up echocardiography examinations.

**Fig. 3:**
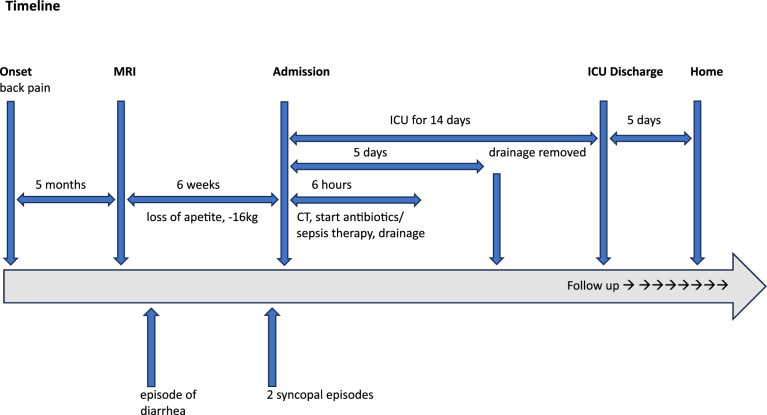
Timeline

Immunologic follow-up investigations did not reveal any predisposing conditions. Antibiotic therapy with amoxicillin and clavulanic acid, along with metronidazole, was continued for a total of 4 weeks. The patient did not experience any relapse or complications after being discharged from the hospital (Fig. 3).

## Discussion

The differential diagnosis of a pericardial effusion includes pericarditis, aortic dissection, myocardial infarction with rupture of the ventricular wall, and malignant disease, among others. Pyopericardium, or purulent pericarditis, is a rare condition, accounting for less than 1% of acute pericarditis [4].

Even if pyogenic liver abscesses have been reported in healthy individuals as in our patient, who had no reported personal or family history of immunodeficiency condition, most reported cases of pyopericardium involve patients with predisposing factors such as immunosuppression, alcohol abuse, chest trauma, or previous pericardial inflammation and fibrosis [4–8]. Moreover, liver abscesses, particularly amoebic and pyogenic types, are more prevalent in low- and middle-income countries due to factors such as poor sanitation, limited healthcare access, and malnutrition [9].

Purulent pericarditis is typically caused by Gram-positive bacteria, particularly *Staphylococci* and *Streptococci* species, through bloodstream infection, whereas *Bacteroides fragilis*, *Hungatella hathewayi*, and *Fusobacterium nucleatum* are rare causes [7, 10]. These species are part of the normal flora of the human intestine [11–14].

The MRI scan conducted 2 months before hospitalization incidentally revealed colitis as an unexpected finding. Hence, we suspected intestinal translocation during an episode of colitis, resulting in a left liver lobe abscess that subsequently ruptured, leading to a pyopericardium by transmigration through the diaphragm. Pyogenic liver abscess is a life-threatening infectious condition, with associated mortality rates ranging from 6% to 14%, commonly caused by either biliary tract diseases (that is, gallstones, malignant obstruction) or bowel diseases causing portal vein pyemia [2, 15]. The rupture of a PLA leading to the formation of a subdiaphragmatic abscess and pyopericardium as a complication has been reported, particularly in cases where the left lobe of the liver is affected [2, 16]. Previous studies have identified *K. pneumoniae*, *E. coli*, and *Acinetobacter* spp., as well as *M. morganii*, *K. oxytoca*, *C. koseri*, *Candida* spp., *Enterococcus* spp., *Peptostreptococcus* spp., and *Proteus* in PLA-associated pericarditis [2]. *Fusobacterium nucleatum* was first reported in purulent pericarditis in 1983 [17]. There are cases of *Bacteroides fragilis* originating from a liver abscess, leading to pericardial effusion after a history of cholangitis up to 4 months prior [11]. While a few other cases of pericarditis caused by the bacterial species *Clostridium* have been published, to our knowledge, this is the first case in which *Hungatella hathewayi* infection was found to be associated with a case of purulent pericarditis [18].

The favorable outcome was based on early and accurate diagnosis and antibiotic as well as interventional therapy and may contribute to avoiding long-term complications such as constrictive pericarditis or aortic mycotic aneurysm. This case highlights the uncommon occurrence of an advanced complication of pyogenic liver abscess and emphasizes the importance of multidisciplinary teamwork.

Although similar cases have been described in immunocompromised or traumatically injured patients, the primary limitation of our case lies in the inability to definitively confirm the suspected cause and entry mechanism. This also limits its generalizability to other cases. However, it underscores the importance of considering such case scenarios, even when established risk factors are not readily apparent.

## Conclusion

Pericardial effusion is a potentially life-threatening complication of a multitude of underlying conditions. Our case of a young and otherwise healthy male patient with a ruptured liver abscess demonstrates that awareness of unusual causes is necessary, especially with fairly non-specific symptoms.

## Data Availability

Sequencing data for the three isolates are available at NCBI GenBank with BioSample accession numbers SAMN30209857, SAMN30209858, and SAMN30209859. Other datasets used in this study are not publicly accessible because they contain identifying patient data. They can be provided by the corresponding author upon reasonable request.

## Abbreviations

PLA: Pyogenic liver abscess
pct: Procalcitonin
CRP: C-reactive protein
ED: Emergency department
